# Breastfeeding after Gestational Diabetes: Does Perceived Benefits Mediate the Relationship?

**DOI:** 10.1155/2017/9581796

**Published:** 2017-03-22

**Authors:** Jordyn T. Wallenborn, Robert A. Perera, Saba W. Masho

**Affiliations:** ^1^Division of Epidemiology, Department of Family Medicine and Population Health, School of Medicine, Virginia Commonwealth University, 830 East Main Street, Suite 821, P.O. Box 980212, Richmond, VA 23298-0212, USA; ^2^Department of Biostatistics, School of Medicine, Virginia Commonwealth University, 830 East Main Street, P.O. Box 980032, Richmond, VA 23298-0032, USA

## Abstract

*Introduction*. Breastfeeding is recognized as one of the best ways to decrease infant mortality and morbidity. However, women with gestational diabetes mellitus (GDM) may have breastfeeding barriers due to the increased risk of neonatal and pregnancy complications. While the prevalence of GDM is increasing worldwide, it is important to understand the full implications of GDM on breastfeeding outcomes. The current study aims to investigate the (1) direct effect of GDM on breastfeeding duration and (2) indirect effect of GDM on breastfeeding duration through perceived benefits of breastfeeding.* Methods*. Prospective cohort data from the Infant Feeding and Practices Study II was analyzed (*N* = 4,902). Structural equation modeling estimated direct and indirect effects.* Results*. Perceived benefits of breastfeeding directly influenced breastfeeding duration (*β* = 0.392, *p* ≤ 0.001). GDM was not directly associated with breastfeeding duration or perceived benefits of breastfeeding. Similarly, GDM did not have an indirect effect on breastfeeding duration through perceived benefits of breastfeeding.* Conclusions*. Perceived benefits of breastfeeding are an important factor associated with breastfeeding duration. Maternal and child health care professionals should enhance breastfeeding education efforts.

## 1. Introduction

Breastfeeding helps infants reach their full health, development, and psychosocial potential [1]. Breastfeeding not only reduces the rate of morbidity and mortality in children [2, 3], but also reduces the likelihood of certain cancers and chronic diseases in mothers [4, 5]. Despite the widespread benefits, approximately half (51.8%) of mothers in the United States breastfeed for six months [6]—the recommended duration according to the American Academy of Pediatrics [7].

Research has demonstrated that a variety of factors including race/ethnicity [8], and Type 1 diabetes [9], impact breastfeeding practices. However, women with gestational diabetes mellitus (GDM) may have an increased risk of breastfeeding for a shorter duration since higher rates of neonatal and pregnancy complications are reported among women with GDM [10]. Moreover, women with GDM may have delayed lactogenesis that could lead to lower rates of breastfeeding [11].

While the prevalence of GDM increases worldwide, it is estimated that up to 14% of pregnancies in the United States (US) are impacted by GDM [12]. Despite recent trends of GDM, research investigating the relationship between GDM and breastfeeding is limited. To the authors' knowledge, two studies have been conducted on GDM and breastfeeding [13, 14], both of which utilized international samples. Results from a retrospective cohort analysis conducted in Ontario reported that women with GDM were less likely to breastfeed (odds ratio (OR) = 0.77; 95% confidence interval (CI) = 0.68–0.87) compared to women without GDM after controlling for potential confounders [13]. Because of the differences in breastfeeding practices between countries and the demonstrated relationship between breastfeeding and infant outcomes for mothers with GDM [15, 16], further research is needed to understand the relationship between GDM and breastfeeding duration in the US.

The current research is supported by the framework of the health belief model (HBM), which asserts that health behavior is dependent upon (1) a desire to avoid illness and (2) a belief that the threat of illness will be prevented through a health action [17]. In addition to these variables, the HBM also incorporates perceived factors such as susceptibility, severity, benefits, and barriers [17]. In the context of breastfeeding, the HBM suggests that women who believe that breastfeeding prevents illness will be more likely to initiate breastfeeding and breastfeed for a longer duration. Consistent with the HBM, a prospective study showed a strong correlation (*r* = 0.455, *p* = 0.0001) between perceived benefits of breastfeeding and breastfeeding practices [18].

**Figure 1:**
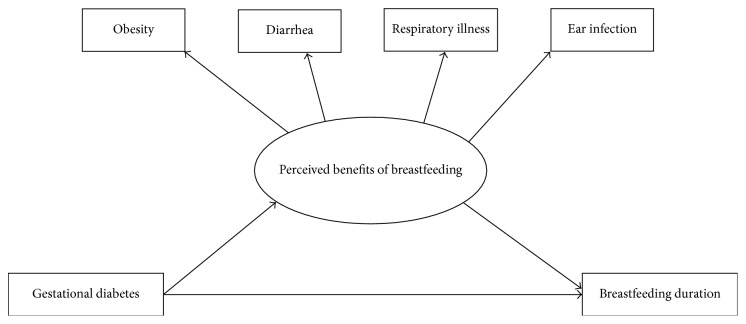
Gestational diabetes, perceived benefits of breastfeeding, and breastfeeding duration conceptual model.

Since women with GDM have to monitor their blood glucose levels [19], they may attend more prenatal care visits compared to women without GDM. This could result in an increased knowledge of breastfeeding benefits due to the increase in available educational interventions and contact with health care providers. Therefore, we hypothesize that perceived benefits are a mediator in the association between GDM and breastfeeding. Despite showing associations between GDM and breastfeeding [13, 14], only two studies of international populations have investigated this relationship. In order to provide insight into the relationship between GDM and breastfeeding outcomes in the US, this study aims to investigate (1) the direct effect of GDM on breastfeeding duration and (2) the indirect effect of GDM on breastfeeding duration through perceived benefits of breastfeeding (Figure 1).

## 2. Materials and Methods

The current study utilized longitudinal data from the Center for Disease Control and Prevention's Infant Feeding and Practices Study II (IFPS II). Data for IFPS II was collected from May 2005 to June 2007 in the US. Participants were identified using a nationally representative consumer opinion panel of households that resulted in a sample of 4,902 women. To be included in the study, mothers and their children must have been in good health, defined as “neither the mother nor the infant could have a medical condition at birth that would affect feeding and that the infant had to have been born after at least 35 weeks' gestation, weigh at least 5 lb, be a singleton, and not have stayed in the intensive care for >3 days [20].” The survey also excluded mothers who were less than 18 years old at the time of the first questionnaire or whose infants later developed a condition or illness that impacted feeding in the first year of life. Information was collected on maternal and child health, infant feeding behaviors, and a mother's diet. Further description on the IFPS II methodology and questionnaires [20] can be found elsewhere.

The exposure and mediator variables were ascertained from the prenatal questionnaire. Gestational diabetes (yes; no), the exposure variable, was based on the survey item: “Have you had gestational diabetes with this pregnancy?” Perceived benefits of breastfeeding, the latent mediator variable, was measured using the survey items: “How strongly do you agree or disagree with the following statements: If a baby is breastfed, he or she will be less likely to (get an ear infection; get a respiratory illness; get diarrhea; become obese).” Participants responded using a five-item Likert scale of strongly agree to strongly disagree. The outcome, breastfeeding duration (continuous), was based upon three postnatal survey questions pertaining to breastfeeding: “Did you ever breastfeed this baby (or feed this baby your pumped milk)?,” “Have you completely stopped breastfeeding and pumping milk for your baby?,” and “How old was your baby when you completely stopped breastfeeding and pumping milk?” If mothers were still breastfeeding at last questionnaire (12 months postpartum) (*N* = 917), the following survey question was asked at the six-year follow-up and was used to determine breastfeeding duration: “How old was your 6-year-old when the following happened? He or she stopped being fed breast milk, including pumped breast milk.”

Various factors were considered as historical confounders for both endogenous and exogenous variables as determined in the literature. These included marital status (married; not married), maternal race (White; Black; Hispanic; other, including Asian/pacific islander), maternal age (continuous), maternal education (less than high school; high school graduate; 1–3 years of college; college graduate), income (less than $20,000; $20,000–49,999; at least $50,000), prepregnancy body mass index (underweight (<18.5 kg/m^2^); normal weight (18.5–24.9 kg/m^2^); overweight (25.0–29.9 kg/m^2^); obese (30.0+ kg/m^2^)), and health insurance or health care plan (yes; no).

Descriptive statistics were used to examine the distribution of data. Structural equation modeling (SEM) was conducted. A single factor model using the indicators for perceived benefits of breastfeeding will be fit using the two-step approach [21]. SEM was the most appropriate approach for examining the association between GDM and breastfeeding because of the ability to simultaneously estimate the direct and indirect mediation effect that GDM has on breastfeeding duration. The two-step approach starts with a confirmatory factor analysis (CFA) followed by a structural model if there is evidence of good fit. The factor loading of ear infection, an indicator variable for the latent factor (perceived benefits of breastfeeding), was fixed to 1 for model identification. Once the CFA was determined to have good fit, then the structural model was developed. Considered fit indices and their prespecified goodness of fit cutoffs included root mean square error of approximation (RMSEA; <0.05), comparative fit index (CFI; >0.90), chi-square test (*p* < 0.05), and weighted root mean residual (WRMR; <1.00). Parameters were estimated using robust diagonally weighted least squares (DWLS) which is the preferred approach for analyzing categorical variables [22]. The mediation effect of GDM on breastfeeding duration through perceived benefits was tested using the indirect effect with a percentile bootstrap confidence interval (CI). Descriptive statistics were calculated using SAS version 9.4 statistical software (SAS, Cary, NC), and SEM analyses were performed in R [23] using the Lavaan package [24].

## 3. Results

The majority of the respondents were white (82.8%), were married (75.5%), had at least a high school diploma (75.3%), and reported initiation of breastfeeding (85.7%). Approximately a quarter (24.8%) reported being overweight, and a third (32.4%) were aged 25–29 years. Over half of participants disagreed or neither agreed nor disagreed that breastfed babies would be less likely to have diarrhea (50.7%) or become obese (63.8%). Maternal characteristics were similar between GDM groups. However, a higher proportion of women who reported having GDM were older and obese (Table 1).

The measurement model showed evidence of good fit (*χ*^2^ (DF = 8) = 86.331; *p* < 0.001; CFI = 0.999; RMSEA (90% CI) = 0.06 (0.049, 0.072); WRMR = 1.617), allowing us to fit the structural model that is saturated. Fit statistics for the structural model are identical to the measurement model as the structural model is saturated. The final model included 2,739 observations. The crude model showed no direct effect of GDM on either breastfeeding duration or perceived benefits of breastfeeding. Similarly, there was no indirect effect of GDM on breastfeeding duration through perceived benefits of breastfeeding (Table 2). However, there was a direct effect of perceived benefits of breastfeeding on breastfeeding duration (*β* = 0.392, *p* ≤ 0.001). After adjusting for marital status, race, education, income, insurance, age, and prepregnancy body mass index, the relationship between knowledge of breastfeeding benefits and breastfeeding duration remained significant while all GDM paths were not significant (Table 3).

## 4. Discussion

The current findings suggest that GDM does not impact breastfeeding duration directly or indirectly through perceived benefits of breastfeeding; however, perceived benefits of breastfeeding are associated with breastfeeding duration. Our finding that perceived benefits are associated with increased breastfeeding is consistent with the HBM and can be supported through the existing, although limited, literature examining the relationship between perceived benefits of breastfeeding and breastfeeding outcomes. A study conducted by Chezem et al. (2003) found a strong correlation between breastfeeding knowledge and breastfeeding duration [18]. Furthermore, Kornides and Kitsantas (2013) found that mothers with greater knowledge of breastfeeding benefits were 11.2 times more likely to initiate breastfeeding and 5.6 times more likely to breastfeed for a longer duration [25]. These findings can be explained using the HBM, which predicts that a health behavior such as breastfeeding is dependent upon the belief that a health action can prevent illness [17].

Even though the current study did not find an association between GDM and breastfeeding duration, these findings are important to disseminate especially in light of current studies that suggest GDM can impact breastfeeding practices. Specifically, a review study investigating Type 2 diabetes and GDM stated that women with GDM have higher rates of pregnancy and neonatal complications that can create barriers to breastfeeding. Further, GDM is more common in obese women which may be an additional barrier to breastfeeding [26]. Various factors such as hormonal response [27], latching challenges [28], body image, and embarrassment have been reported to influence breastfeeding practices among overweight or obese women [29].

To the authors' knowledge, this is one of the first studies to closely examine the pathway between GDM and breastfeeding duration. This study utilized a prospective cohort design that allowed clear temporal sequence. Findings from the current study make a valuable contribution to the literature by demonstrating the direct effect of perceived benefits on breastfeeding duration and by disputing the limited research examining GDM and breastfeeding. Lastly, research on GDM is especially timely and salient due to current GDM trends and a national push to increase breastfeeding rates.

Despite its strengths, this study is not without limitations. Data from IFPS II may not be generalizable due to participants having a higher mean education level, being of older age, being white, being more likely to have middle income, being employed, being less likely to smoke, and having fewer children compared to a representative sample from the National Survey of Family Growth [20]. However, the homogenous population reduces potential bias from residual confounding. Social desirability bias may influence mothers to overestimate their breastfeeding duration leading to nondifferential misclassification bias, which could bias the estimate towards from the null—however, previous research has demonstrated that self-reported breastfeeding duration is a reliable measure. Lastly, potential confounding factors such as substance abuse, breastfeeding self-efficacy, and perceived milk supply were not available in the dataset and could not be assessed.

## 5. Conclusions

Our results suggest that GDM does not have a direct or indirect effect on breastfeeding duration; however, perceived benefits of breastfeeding have a direct effect on breastfeeding duration. Healthcare and public health professionals can utilize this information to strengthen current and future interventions by educating women about the benefits of breastfeeding which may increase breastfeeding rates. Due to inconsistent findings in the few studies investigating GDM and breastfeeding, further research is warranted.

## Figures and Tables

**Table 1:** Distribution of maternal characteristics by study population and gestational diabetes mellitus^a^.

Characteristic	Overall	GDM	No GDM	*χ* ^2^
	Percent	Percent (*N* = 310)	Percent (*N* = 4134)	(*p* value)
*Age*				*<0.0001*
18–24 years	28.3	17.2	28.2	
25–29 years	32.4	27.9	33.0	
30–34 years	25.0	27.0	25.3	
35–52 years	14.4	27.9	13.5	
*Marital status*				0.43
Married	75.5	77.9	75.8	
Not married	24.5	22.1	24.2	
*Maternal race*				0.12
White, non-Hispanic	82.8	79.5	81.6	
Black, non-Hispanic	5.3	4.4	6.4	
Hispanic	6.7	9.1	6.8	
Other	5.1	7.1	5.2	
*Maternal education*				0.80
Less than high school	4.8	4.4	4.5	
High school	19.9	21.9	19.4	
1–3 years of college	41.1	39.3	41.3	
College graduate	34.2	34.4	34.8	
*Income*				0.55
<$20,000	15.6	15.8	16.1	
$20,000–$49,999	41.4	40.7	43.4	
≥$50,000	43.0	43.6	40.5	
*Health insurance*				0.63
Yes	94.7	95.4	94.8	
No	5.3	4.6	5.2	
*Prepregnancy BMI*				*<0.0001*
Underweight (<18.5 kg/m2)	5.3	1.7	5.5	
Normal weight (18.5–24.9 kg/m2)	46.3	26.7	47.9	
Overweight (25.0–29.9 kg/m2)	24.8	26.1	24.8	
Obese (30.0+)	23.6	45.5	21.8	
*Breastfeeding duration *				*0.06*
Never breastfed	14.3	20.4	14.2	
Breastfed less than 6 months	42.7	39.8	45.0	
Breastfed 6 or more months	43.0	39.8	38.0	
*Breastfeeding prevents diarrhea* ^b^				0.79
Strongly disagree	5.7	6.1	5.7	
Somewhat disagree	8.9	9.0	9.0	
Neither agree or disagree	36.1	38.4	35.4	
Somewhat agree	26.6	26.1	27.0	
Strongly agree	22.8	20.3	22.8	
*Breastfeeding prevents obesity* ^b^				0.48
Strongly disagree	10.9	13.6	10.7	
Somewhat disagree	10.1	8.4	10.3	
Neither agree or disagree	42.8	43.2	42.5	
Somewhat agree	19.7	19.0	19.7	
Strongly agree	16.5	15.8	16.9	
*Breastfeeding prevents ear infections* ^b^				0.16
Strongly disagree	5.3	6.8	5.2	
Somewhat disagree	6.5	9.4	6.3	
Neither agree nor disagree	25.4	23.4	25.0	
Somewhat agree	31.2	29.2	31.5	
Strongly agree	31.6	31.2	32.0	
*Breastfeeding prevents respiratory illness* ^b^				0.45
Strongly disagree	5.1	6.5	5.0	
Somewhat disagree	6.0	7.8	6.0	
Neither agree nor disagree	25.6	25.3	25.1	
Somewhat agree	32.5	29.9	33.1	
Strongly agree	30.7	30.5	30.7	

GDM = gestational diabetes mellitus, BMI = body mass index.

^a^Not all percentages sum to 100% due to rounding.

^b^The following category is from an IFPS II survey question asking, “How strongly do you agree or disagree with the following statements: If a baby is breastfed, he or she will be less likely to (get an ear infection; get a respiratory illness; get diarrhea; become obese)”.

**Table 2:** Parameter estimates of the indirect effect of GDM on breastfeeding duration through perceived benefits of breastfeeding.

Parameter	Estimate	Bootstrap CI
*Crude model*		
Indirect effect of GDM on breastfeeding duration through breastfeeding benefits	0.52 (0.59)	−0.62–1.69

*Fully adjusted model* ^a^		
Indirect effect of GDM on breastfeeding duration through breastfeeding benefits	0.01 (0.64)	−1.16–1.19

GDM = gestational diabetes mellitus; CI = confidence interval.

^a^Adjusted for marital status, race, education, income, insurance, age, and prepregnancy body mass index.

**Table 3:** Parameter estimates of direct effects.

Parameter	Estimate (standard error)	*Z*-value	*p*
*Crude model*			
Direct effect of GDM on breastfeeding duration	1.18 (1.44)	0.82	0.411
Direct effect of breastfeeding benefits on breastfeeding duration	1.98 (0.09)	21.18	<0.0001
Direct effect of GDM on breastfeeding benefits	0.26 (0.29)	0.89	0.373

*Fully adjusted model* ^a^			
Direct effect of GDM on breastfeeding duration	1.27 (1.51)	0.85	0.398
Direct effect of breastfeeding benefits on breastfeeding duration	1.91 (0.10)	19.78	<0.0001
Direct effect of GDM on breastfeeding benefits	0.01 (0.32)	0.02	0.988

GDM = gestational diabetes mellitus; CI = confidence interval.

^a^Adjusted for marital status, race, education, income, insurance, age, and prepregnancy body mass index.
